# *QuickStats:* Percentage of Mothers with Gestational Diabetes,* by Maternal Age — National Vital Statistics System, United States, 2016 and 2021

**DOI:** 10.15585/mmwr.mm7201a4

**Published:** 2023-01-06

**Figure:**
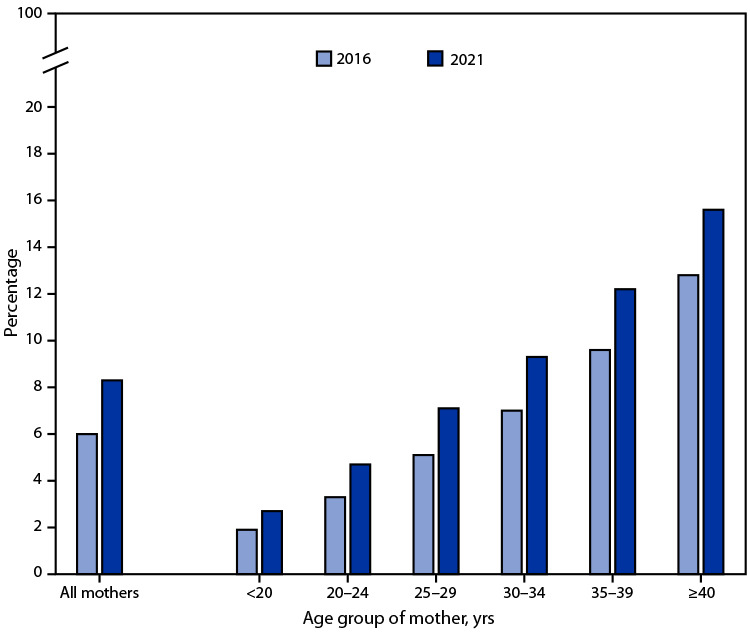
The percentage of mothers giving birth who received a diagnosis of diabetes during pregnancy (gestational diabetes) increased from 6.0% in 2016 to 8.3% in 2021. Increases in gestational diabetes were seen in each maternal age group, and rates rose steadily with maternal age; in 2021, the rate for mothers aged ≥40 years (15.6%) was nearly six times as high as the rate for mothers aged <20 years (2.7%).

**Source:** National Center for Health Statistics, National Vital Statistics System, Natality Data. https://www.cdc.gov/nchs/nvss/births.htm

For more information on this topic, CDC recommends the following link:  https://www.cdc.gov/pregnancy/diabetes.html

